# Direction based Hazard Routing Protocol (DHRP) for disseminating road hazard information using road side infrastructures in VANETs

**DOI:** 10.1186/2193-1801-3-173

**Published:** 2014-04-02

**Authors:** M A Berlin, Sheila Anand

**Affiliations:** Department of Computer Science and Engineering, R. M. D Engineering College, RSM Nagar, Kavaraipettai, Chennai, 601206 Tamil Nadu India; Computer Studies, Rajalakshmi Engineering College, Thandalam, Chennai, 602105 Tamil Nadu India

**Keywords:** Road hazards, RSUs, Highways, Direction based, Selective forwarding, Hazard message, Verification of hazard message

## Abstract

This paper presents Direction based Hazard Routing Protocol (DHRP) for disseminating information about fixed road hazards such as road blocks, tree fall, boulders on road, snow pile up, landslide, road maintenance work and other obstacles to the vehicles approaching the hazardous location. The proposed work focuses on dissemination of hazard messages on highways with sparse traffic. The vehicle coming across the hazard would report the presence of the hazard. It is proposed to use Road Side fixed infrastructure Units for reliable and timely delivery of hazard messages to vehicles. The vehicles can then take appropriate safety action to avoid the hazardous location. The proposed protocol has been implemented and tested using SUMO simulator to generate road traffic and NS 2.33 network simulator to analyze the performance of DHRP. The performance of the proposed protocol was also compared with simple flooding protocol and the results are presented.

## Introduction

Vehicular ad hoc network (VANET) is an emerging technology for Intelligent Transportation Systems (ITS). VANETs are formed by vehicles which are equipped with wireless communicating devices capable of transmitting information. VANETs are primarily used for two types of applications; safety and non-safety applications (Berlin and Sheila 2011). Safety applications focus on risk free travel. Safety messages include road hazard or obstacle warning, accident warning, curve warning, traffic jam, and other driver related warning such as drunken driving, emergency braking, lane change etc. Communicating safety messages in advance to the vehicles approaching the hazardous zones would enable the drivers to take suitable safety measures. Non-safety applications focus on providing timely information for the comfort of the driver and passengers that includes nearest restaurant information, gas filling stations, accessing internet, weather conditions and entertainment etc.

Messages to vehicle drivers can be categorized into two types: delay tolerant and delay sensitive messages (Nawaz and Ali 2011). Messages related to road hazard or accident warning are delay sensitive messages as timely dissemination of these messages would help to avoid accidents, collisions and traffic jam (Boto and Weber 2011). Comfort applications are examples of delay tolerant messages (Yuh- Shyan et al. 2010). The Federal Communication Commission (FCC) has allotted 75 MHz of frequency bandwidth at 5.9 GHz of Dedicated Short Range Communication (DSRC) spectrum for the transmission of safety and hazard related messages using Control Channel (CCH) and Service Channels (SCHs) (Saleh et al. 2007; Yi et al. 2008).

VANETs support two types of communication: Vehicle-to-Vehicle (V2V) and Vehicle-to-Road side units (V2R) (Boukerche et al. 2008). In V2V communication, the vehicles are used as relay nodes to forward hazard messages between the vehicles. In V2R communication, the vehicles or Road Side Units (RSUs) may act as relay nodes to transmit the information to the vehicles. In V2V communication, the vehicles travelling on the road would act as forwarders to transmit the messages.

This paper focuses on transmission of fixed road hazard messages in highways with the use of smart RSUs. Fixed road hazards would include tree or boulder fall. In highways with sparse traffic, the vehicle encountering the hazard is likely to be the primary source of information about the hazard. V2V communication would not be a feasible option as the vehicular traffic is not dense. Hence, V2R communication has been proposed for timely delivery of the road hazard messages. Vehicles which receive such hazardous message in advance would be able to take appropriate measures, like re-routing, to avoid the hazardous location.

### Related work

Much of the research work has been focused on developing protocols and methodologies to disseminate safety and road hazards using vehicle-to-vehicle communication. Vehicles are used as forwarder nodes for transmission of these messages to other vehicles approaching the danger zones. This pre-supposes that the vehicular traffic is sufficient to ensure transmission. V2V communication would not be possible in areas where vehicular traffic is light. However, the concepts of store and forwarding and selective forwarding are important and need to be considered while designing safety message dissemination protocols for highway scenarios with sparse population of vehicles. Hence in this section, we first discuss work related to protocols for safety message propagation. Since we propose the use of RSUs for our protocol, we also discuss related work that uses RSUs for disseminating safety related messages.

Songnan Bai et al. have presented a Vehicular Multi-hop broadcast Protocol (VMP) for the dissemination of safety messages using other vehicles as forwarder nodes based on the neighboring information received by hello messages (Songnan et al. 2009). The vehicle which encounters a dangerous zone can transmit alert message with two forwarder details. A node with the smallest forwarding delay will rebroadcast the alert message. Obviously, the farthest node will act as a forwarder node. The authors have compared the performance of VMP with other broadcast suppression protocols such as DDB (Marc et al. 2006), slotted 1 and weighted P (Wisitpongphan et al. 2007), flooding protocols. Do-hyeon Lee et al. have proposed a selective alert message forwarding scheme for reducing the number of nodes involved in re-broadcasting (Do-hyeon et al. 2010). Each node maintains a forwarding table to select the best forwarder based on the positional information it has received from its neighbors. The forwarding node is selected based on the direction of movement of the node, distance, and relative velocity. V2V communication is used for transmitting safety related messages and they have compared the performance of this scheme with other suppression schemes such as weighted p-persistence, DDB, and slotted 1-persistence. They were able to substantially reduce the duplicate transmissions as compared to other suppression schemes.

Natarajan Meganathan et al. have presented a Risk Notification Messages (RNMs) protocol for disseminating risk zone messages to the neighboring vehicles (Natarajan and Skelton 2010). They have assumed that the RNMs messages are generated by police vehicles or other sensors from the risk zone and transmitted to the vehicles (nodes) which move towards the risk zone. Each vehicle calculates the rebroadcast time based on the distance between the receiver and the transmitter node and the maximum transmission range of the nodes. The authors have compared their work with simple flooding of messages. Pakornsiri Akkhara et al. have proposed new broadcasting approaches for dissemination of alarm messages on highways (Pakornsiri et al. 2008). It is assumed that all vehicles are equipped with sensors, transceivers and Global Positioning System (GPS) receivers. High priority is given to the farthest vehicle which has less waiting time for broadcasting alarm messages. The number of rebroadcasts is reduced based on the notification flags received by all nodes. The authors have studied their work with reference to straight highway scenario and planned to extend their work to apply to roads with various shapes.

Yao-Tsung et al. have proposed a broadcast algorithm called Position-based Adaptive Broadcast (PAB) for transmitting emergency messages to the rear vehicles on highways (Yao-Tsung and Li- Der 2008). It assumes that each vehicle is equipped with a GPS receiver to know its positional information. The source vehicle which senses the emergency event will broadcast the safety message. Then the farther nodes of the transmission range of source vehicle will rebroadcast the safety messages. The authors have proven the dissemination of emergency messages with low latency and minimum number of retransmissions. They have also pointed out that the proposed algorithm needs improvement to apply for other applications such as tollbooth scenario and active emergency warning scenario. Yu-Tian Tseng et al. have proposed a Vehicle-Density based Emergency Broadcast (VDEB) scheme for broadcasting emergency messages (Yu-Tian et al. 2010). They have attempted to find a solution for high overhead and long delay of the delivery of the emergency messages on highways. Each vehicle exchanges hello messages with other vehicles to maintain a neighbor table. When an emergency event occurs, the current forwarder divides the transmission range into number of rings. The vehicle which is in outer ring has zero waiting time and can rebroadcast the emergency message.

We also explore the recent work that use selective forwarding and carry and forward approaches used for disseminating safety related messages. Ramon S. Schwartz et al. have presented a Simple and Robust Dissemination (SRD) protocol for broadcasting hazard and event-based messages in a specific direction (Schwartz et al. 2011). The authors have assumed that there are no road side infrastructures on highways. Hence the vehicles have to detect and generate the hazard messages. The vehicle which is at the tail of the cluster will store, carry and rebroadcast the hazard message. The authors have modified the slotted 1 persistence which is presented in (Schwartz et al. 2010). They have used this suppression technique on dense networks and the store-carry-forward technique on highways. Mahmoud Abuelela et al. have proposed a data dissemination approach called SODA – a Smart Opportunistic Data dissemination Approach to disseminate packets in disconnected roads on highways (Mahmoud and Stephan 2009). Vehicles exchange beacon messages to form clusters and maintains its position, speed and neighbors details which belong to other clusters. The node which is transmitting the message would carry it without forwarding to its neighbors based on its relative speed with respect to its neighbors for ensuring no wastage of bandwidth.

Nicolas Cenerario et al. have described a dissemination protocol for transmitting information related to different events such as an accident, an emergency braking, an available parking slot, etc. to vehicles using vehicle-to-vehicle communication (Cenerario et al. 2011). Encounter probability (EP) has been used to represent the probability of the vehicle to meet certain event which is relevant to it. If EP value is high for a vehicle for some event, then the event would be considered as relevant for that vehicle. The authors have compared this dissemination protocol with simple flooding and periodic flooding protocols. They have obtained limited overhead and could see that all the vehicles receive the interesting events before they reach the event. The authors have planned to extend their work to apply in real complex scenarios with high vehicular density and to use available infrastructures for communication.

We now look at some related work that use RSUs for disseminating safety related messages. Kai Liu et al. have presented a RSU-Assisted Multi-channel Coordination MAC (RAMC) protocol to provide safety and non-safety communication using RSUs (Kai et al. 2009). Each RSU monitors the control and safety channels simultaneously to receive and analyze the hazardous conditions to perform aggregation periodically and warn the other related vehicles. The authors have not specified how the hazard is detected but have assumed that the information is transmitted on the safety channel. The authors have concluded that the RAMC protocol performs well in dense network. Sok-Ian Sou and Ozan Tonguz have analyzed VANET connectivity on highways and have tried to determine the minimum number of RSUs that need to be deployed on the road (Sou and Ozan 2011). The authors have derived the analytical model for determining the connectivity probability of RSUs and vehicles. They have found that the numbers of re-healing nodes are reduced to 68% when there are RSUs on highways.

Christian Lochert et al. in their work have investigated how a VANET-based traffic information system can overcome the two key problems of strictly limited bandwidth and minimal initial deployment using RSUs and data aggregation (Christian et al. 2008). They have proposed a genetic algorithm to identify good positions for static road side units.

Hence, it can be seen that RSUs provide a feasible option for disseminating road safety messages and provide a reliable alternate to V2V communication, especially, in areas where the vehicular traffic is sparse. Concepts of store and selective forwarding can reduce the network load and improve reliability.

### DHRP protocol description

We extend our earlier work to propose and present a Direction based Hazard Routing Protocol (DHRP) for delivering road hazard information to the vehicles travelling on highways (Berlin and Sheila 2012). Road hazards make driving unsafe and the focus of this work is the dissemination of hazard messages related to fixed hazards or obstacles on the road. Fixed road hazards includes tree fall, boulder fall, landslide, snow heaps and road maintenance blocks. Vehicle drivers receiving the advance warning message can take suitable safety measures like re-routing to avoid the hazards.

Dissemination of hazardous message on highways where the traffic is sparse is a challenging task in VANET. In urban areas, the density of vehicles is generally very high, and V2V communication would be a viable option for disseminating road safety messages. The civic authorities would be physically monitoring the city roads to ensure that they are clean and hazard free. But on highways, intimating road hazard information to the highway authority may itself be a challenging task if the traffic is sparse. The vehicle which encounters the road hazard would have to report the presence of the hazard. The vehicle can store the message and disseminate to other vehicles approaching the hazard location (Ozan et al. 2007). However, the direction of travel of the vehicle which is carrying the hazard message may not be the direction to which it has to forward the message.

The focus of the proposed work is road hazard information dissemination on highways where the traffic is sparse. As the traffic is assumed to be sparse, V2V communication would not be a feasible solution. RSUs deployed on highways can act as forwarder nodes to transmit hazard related messages to the vehicles moving in the direction of the located hazard. RSUs are smart infrastructure based devices which are deployed on the road side that are capable of communicating with the vehicles (Yuwei et al. 2010). RSU has sufficient storage capacity, capability of computing and communicating through wireless equipments and provide a very reliable communication model. RSUs can be either infrastructure based or be deployed on an ad-hoc basis (Sou and Ozan 2011). Infrastructure based RSUs would be interconnected using fiber or wireless links but the cost of deployment and maintenance would be high. In ad-hoc based model, RSUs communicate using wireless communication and the cost of deployment would be more reasonable.

The protocol proposes the deployment of RSUs relatively close to each other with capability of communicating with its neighboring RSUs and with the vehicles travelling on the highway. The communication range (CR) of RSUs would extend to single and multi-lane highways so that all the lanes are within its communication zone. As RSUs are stationary infrastructure, the use of RSUs improves the reliability of the transmission of messages. The messages can also be broadcast by the RSUs with minimal delay to the vehicles moving towards the hazard location. Hence, the use of RSUs can guarantee the reliable and speedy delivery of road hazard messages to vehicles.

The proposed protocol also addresses the key issue of verification of the hazard message, which is explained in detail in the next section. RSUs would verify the received hazard message for correctness and reject messages found to be false. Messages verified to be correct can be broadcast to vehicles approaching the hazard. We propose selective transmission of hazard message based on the location of hazard and the direction in which the vehicles are travelling. This would considerably reduce the network load and enhance the reliability of transmission.

For VANET communication, each vehicle is equipped with a wireless communication device called On-Board Unit (OBU). It is assumed that OBU is integrated with a GPS receiver and road hazard detecting equipment for detecting road hazards. We assume that the vehicles are capable of detecting different types of hazards both during the day and night and under all weather conditions. The actual work of hazard detection and categorization of hazard type would be addressed as future work. We propose that RSUs are deployed on the highways and are capable of communicating with its neighboring RSUs and vehicles travelling on the highway. The contributions of the proposed protocol may be summarized as follows:

1. The proposed DHRP protocol is suitable for highways where vehicular traffic is sparse.

2. It is proposed to use RSUs for achieving reliable transmission and speedy delivery of the Hazard Message (HM) to vehicles approaching the hazard location.

3. RSUs would also verify the correctness of the received hazard message and reject false messages.

**Figure 1:**
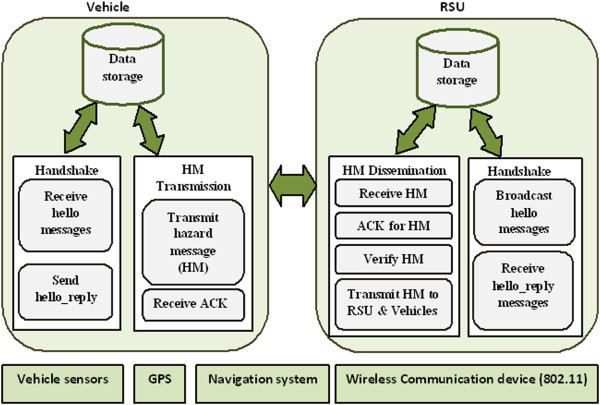
**DHRP protocol architecture.**

The architecture of our proposed protocol is shown in Figure 1.

The Handshake protocol component deals with establishing communication between RSU and the vehicles travelling on the highway. RSUs will periodically broadcast *hello_message* to the vehicles travelling in its transmission range which contains < RSU_ID, GPS location of RSU, Timestamp>. The vehicles entering the transmission range of RSU would receive the *hello_message* and send a *hello_reply* message giving its ID, location, direction of travel, date, timestamp and speed to the RSU from which it has received the *hello_message*.

RSU would maintain a table ‘T’ to store the vehicle details. The information in ‘T’ would be used for processing of the hazard messages received from the vehicles as explained later. Since this proposed work considered sparse traffic on highways, it would be possible for RSUs to update and maintain the vehicle information in the table ‘T’. It is assumed that RSUs and vehicles would have a unique identifier (ID) to identify them. The information stored in ‘T’ would include vehicle ID, vehicle GPS location, direction of travel and speed.

As a vehicle moves out of the transmission range of RSU, the entry of the particular vehicle would be removed from the table ‘T’. This is done in the following manner. Let the travelling speed of the vehicle V stored in ‘T’ be ‘S’, the time at which *hello_reply* message received from particular vehicle be ‘t_r_hrep_’, the current time be ‘t_c_’ and the transmission range of RSU be ‘T_range_’. The distance ‘d_v_’ travelled by the vehicle during the time (t_c_ – t_r_hrep_) is calculated as,

If (d_v_ > T_range_), then RSU would remove the information of the vehicle V from ‘T’.

**Figure 2:**
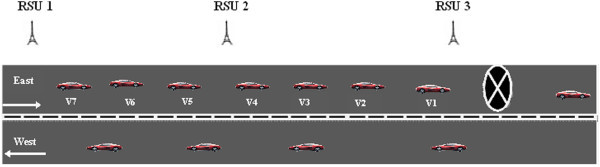
**Hazard at forward lane.**

When a vehicle encounters a road hazard, *H*_*r*_, it generates and transmits a *HM* to the RSU from which it has received the last *hello_message*. The *HM* would include the key information : <*Source vehicle ID, hazard location, hazard_type, timestamp, RSU_ID>.* The protocol is further explained by considering the scenario given in Figure 2 where the highway is a straight road with two lanes and vehicles travelling in the forward and opposite directions.

One of the main design considerations of our DHRP protocol is to drive the *HM* in the specific direction of vehicles approaching the hazardous location. The direction where the hazard is present is considered as the forward lane (F_L_) and the other lane is taken as the opposite lane (O_L_). Our proposed protocol, DHRP, would follow the steps outlined below for selective transmission of *HM* on highways with two lanes as depicted in Figure 2.

- When the vehicle v_1_ detects *H*_*r*_ along its travelling path, it generates a *HM* and broadcasts the message to the nearest RSU which is RSU3 from which it would have received the last *hello* message.
- RSU which receives the *HM* would respond by sending an ACK message. This enables the vehicle to ensure that *HM* has been received by RSU. The vehicle can therefore stop sending the hazard message.
- RSU3 will wait for ‘*t*’ seconds before transmitting the received *HM* to RSU2. It uses this time slot to verify the validity of the received messages and reject false messages. The verified *HM* would be then transmitted to the vehicles in its range and also to its immediate neighbor RSU2 and then on to RSU1 and so on.
- The vehicles which receive the *HM* can take corrective action, for example, re-routing, to avoid the hazard location. If the road/track in which the vehicle is travelling is not blocked; it can ignore the *HM* and proceed as normal. Since the vehicles will not rebroadcast the hazard information to its neighboring vehicles, the network load is highly reduced.

**Figure 3:**
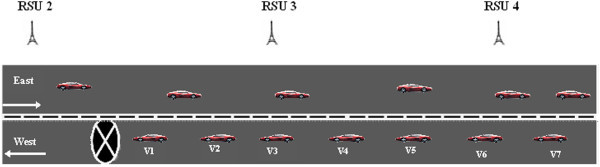
**Hazard at other lane.**

We now consider the case where the hazard is located on the other lane as shown in Figure 3. Vehicle v_1_ is travelling in the west bound direction would detect the *H*_*r*_. The vehicle would generate a *HM* and transmits to the nearest RSU, which is RSU2. RSU2 would verify the *HM* and transmit to RSU3 and RSU4.

**Figure 4:**
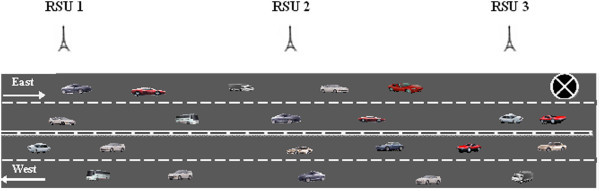
**Single lane blocked on a multilane highway.**

Highways generally have multiple lanes in either direction for vehicular traffic. We now look at a 4-lane traffic scenario and explain the steps to be followed by DHRP to communicate the messages to relevant vehicles. We explain with two cases; the hazard partially blocks the road in the F_L_ and fully blocks the road in the F_L_. Figure 4 depicts a road scenario where the road is partially blocked in a multilane highway.

The F_L_ (east bound direction) consists of two lanes for vehicles to travel from west to east. The *H*_*r*_ blocks only a single lane. Hence, vehicles can proceed further in that direction by changing to the free lane. However, this would cause vehicles to slow down and cause a traffic jam. Vehicles coming across the *H*_*r*_ would generate and transmit the *HM* to RSU3. RSU3 would transmit to RSU1 through RSU2. Vehicles can re-route to avoid the traffic jam or can choose to proceed in the same direction depending on the location of their destination.

**Figure 5:**
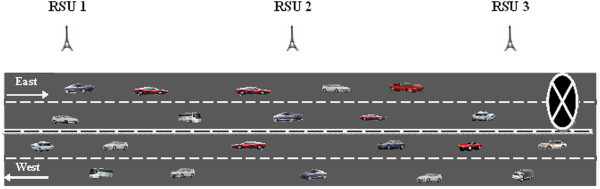
**All lanes in a particular direction blocked on a multilane highway.**

We next look at a scenario where the entire set of lanes in a particular direction is blocked as shown in Figure 5.

**Figure 6:**
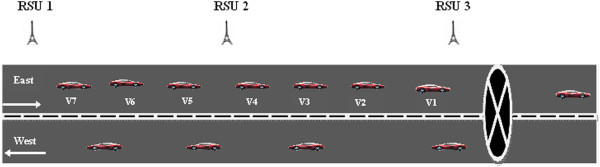
**Road is fully blocked.**

In this case, vehicles cannot proceed further along the highway as all the lanes are blocked. Vehicles have to re-route to avoid the road block. RSU3 will receive the *HM* and transmit to RSU1 through RSU2. Furthermore, we look at the scenario shown in Figure 6, where the road is fully blocked in both the lanes (i. e F_L_ and O_L_). In this case, the vehicles cannot move ahead in both the lanes. Hence the vehicles which are travelling in east bound direction would transmit the *HM* to RSU3 which in turn transmits the message to RSU1 through RSU2. Vehicles travelling in the west bound direction would transmit the hazard message to RSU3 which in turn would transmit to other RSUs towards east.

### Transmission and stopping of *HM* by vehicles

**Figure 7:**
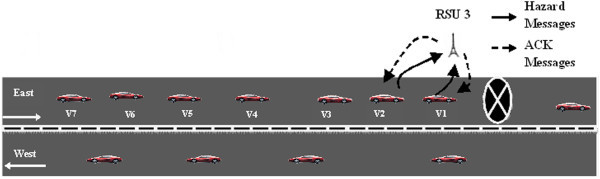
**Stop** ***HM*** **based on ACK message from RSU.**

Vehicles which detect *H*_*r*_ would transmit the *HM* till it receives an acknowledgement (*H*_*ACK*_) from the RSU to which the vehicle sent the *HM*. For example in the scenario shown in Figure 7, vehicle v1 would first detect the *H*_*r*_ and transmits hazard information to RSU3 till it receives an *H*_*ACK*_ from RSU3. This assures the vehicle that RSU has received the hazard message and hence would stop sending *HM*.

**Figure 8:**
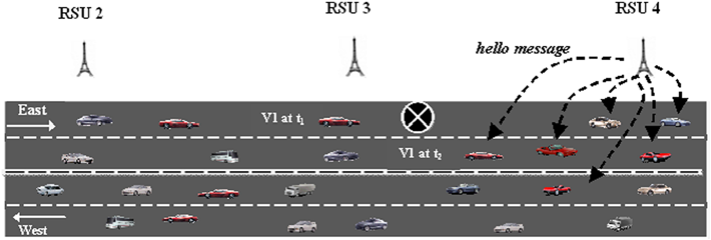
**Stop** ***HM*** **based on** ***hello_message.***

However, if the road is not completely blocked the vehicle would be able to crossover to another lane and proceed further in its intended direction of travel. It would then go out of range of the RSU to which the hazard message was sent. Therefore, the concerned vehicle would stop sending the *HM* when it receives a *hello_message* from another RSU. In the scenario given in Figure 8, vehicle v1 detects the presence of *H*_*r*_ at time *t*_*1*_ and transmits *HM* to RSU3. Since the road is not fully blocked, there is a possibility for the vehicles to travel further in the same direction. So, at time *t*_*2*_ the vehicle can select the other lane and travel in the transmission range of RSU4. At this time the vehicle would receive *hello_message* from RSU4 and start sending the *HM* to RSU4 from which it would receive the *H*_*ACK*_.

### Verification of received hazard messages by RSU

**Figure 9:**
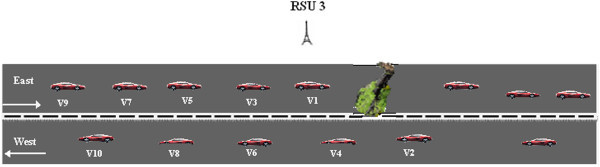
**Verification of hazard message.**

Verification helps to identify and ignore false messages. RSU would broadcast its *hello_message* and receive the *hello-reply* message from the vehicles within its transmission range. All reply messages are analyzed and stored in a vehicle table at the RSU. The details stored would include *vehicle_ID, GPS location of vehicle, current speed* and the *direction of travel of vehicle*. When the RSU receives a *HM* from a vehicle it would first verify the correctness of the received message. The RSU would look up its table ‘T’*,* to calculate how many vehicles are likely to send the same message during the time slot ‘*t*’. It would wait for time ‘*t*’ and check the number of messages it has received. If the number of messages received exceeds a pre-fixed threshold, then it would assume that the *HM* is correct. We explain the verification process with a sample scenario given in Figure 9.

Vehicles v_1_ – v_10_ are within the transmission range of RSU3 and we can assume that it would have received the *hello-reply* message from these vehicles and updated the vehicle table with details of *vehicle_ID, GPS location of vehicle*, *current speed and the direction of travel*. When RSU3 receives a *HM* from v_1_, it would look up the location and other details of v_1_ from *T*. From the Table *T*, it can determine that vehicles travelling in the same direction as v_1_ are v_3_, v_5_, v_7_ and v_9_. It would calculate the likely time that would be taken by these vehicles, starting from v_3_ to reach v_1_ and send the same *HM*. Hence the time taken ‘*T*_*HM(i)*_’ to send the same *HM* from vehicle v_i_ can be calculated based on the distance to be travelled to reach the location of v_1_, the time ‘*t*_*r*_’ taken to identify hazard, and the time ‘*P*_*t*_’ taken by the *HM* to reach RSU3 is,

The distance ‘*d*’ between a vehicle and v_1_ is calculated using the well known *Haversine formula* (Ivis 2006)*.* The time to reach location of v_1_ can be calculated from the speed of the vehicle. Moreover the total time taken ‘*t*’ to receive the same *HM* from ‘*N*’ vehicles which are in the transmission range of RSU3 can be calculated as,

The verification time slot ‘t’ would vary depending upon the speed ‘S’ of the vehicle approaching hazard location which are within the transmission range of RSU. If the speed of the vehicles is high, the verification time slot would be less. During the time slot *‘t’*, let us assume that RSU3 should have received messages from vehicles v_3_, v_5_ and v_7_. It checks the number of actual messages received with the messages that it should have received, which in this example is 4. If the threshold value, *T*_*thresh*_, is set as 50%, then RSU3 would accept that the *HM* as correct, if it receives at least 2 messages, one of which is v_1_ and the others could be from any or all of the vehicles v_3_, v_5_ or v_7_. Else the message is rejected as false and HM is not broadcasted to the other vehicles.

If there are no other vehicles travelling on the road, and v_1_ is the only vehicle, then RSU would wait for another vehicle to also report the hazard before assuming that the message is correct. Since there are no other vehicles travelling on the road, the time delay in broadcasting the hazard information would be acceptable.

### Distribution by RSU

HM verified as correct by RSU would be broadcast to all vehicles within its transmission zone. RSU would also send the HM to neighboring RSU to warn vehicles approaching the hazard location. For instance, with reference to Figure 4, it would be RSU2. RSU2 can then further propagate the message to other RSUs and vehicles in the said direction. The hazard message could be propagated to all RSUs, preceding the road hazard, till a major road intersection is reached. It would be then possible for the vehicles to take alternate routes to avoid the road hazard.

### Implementation and results

The proposed protocol has been verified using SPIN model checker (Berlin and Sheila 2013). Formal verification is a mathematical approach that is used to model the possible behavior of the protocol. The protocol is validated to identify design errors and verify the model properties defined for the protocol.

The performance of the proposed DHRP protocol was tested using simulation. The protocol was implemented and tested using SUMO (Kun-Chan and Chien-Ming 2008) and NS 2.33 (Teerawat and Hossain 2009). The traffic patterns were simulated using SUMO and a .tcl file was created. The .tcl file was taken as input to NS2 for analyzing the performance of DHRP. Traffic models were simulated for both single lane and four lane highways. Blocks which covered single and double lanes were simulated. IEEE 802.11 was used as medium access protocol (MAC). The recommended radio transmission range of RSUs for effective communication is 250m (Tatsuaki et al. 2006). The transmission range determines the number of RSUs that would need to be deployed. The simulation parameters used are given in Table 1.

**Table 1:** **Simulation parameters used in NS2**

Name of parameters	Value
**Number of RSUs used**	4
Traffic density	5 to 20 vehicles
Protocol	DHRP
Hello interval	5 ms
Road scenarios	Single lane and multilane
Transmission range	250 m
Medium access	MAC
Simulation time	400 ms
Road block	Single and double lane
Vehicle speed, S	40 km/hr
Interspacing between vehicles	20 m

If the transmission range is high, the number of RSUs that would need to be deployed would be less and if the transmission range is low, the number of RSUs that need to be deployed would be more. The performance of the protocol with respect to transmission range of 250 m was studied. Timers were used to generate the periodic hello message from RSU. Since the proposed work is with respect to sparse traffic, the performance of DHRP protocol was tested with density of 5, 10, 15 and 20 vehicles. The behavior of the proposed protocol was analyzed for various road block scenarios on highways and the results are presented.

**Figure 10:**
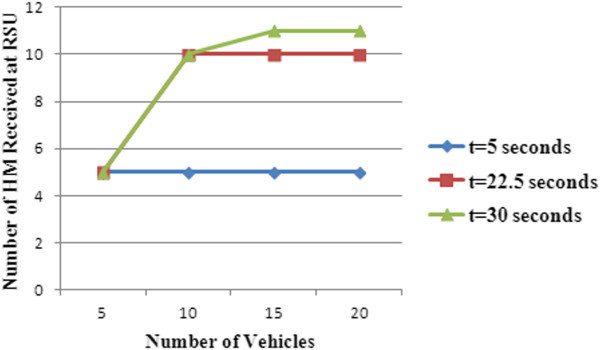
**Count of hazard messages received at RSU.**

Figure 10 shows the count of hazard messages received at RSU for different verification time slots ‘t’ and vehicular density in hazard zone.

It should be noted that verification time slot is calculated based on the speed of the vehicles and with increase in the count of vehicles travelling towards the hazard, the time slot is likely to be higher. It can be seen from the graph that the number of HM received at RSU increases as verification slot ‘t’ increases.

**Figure 11:**
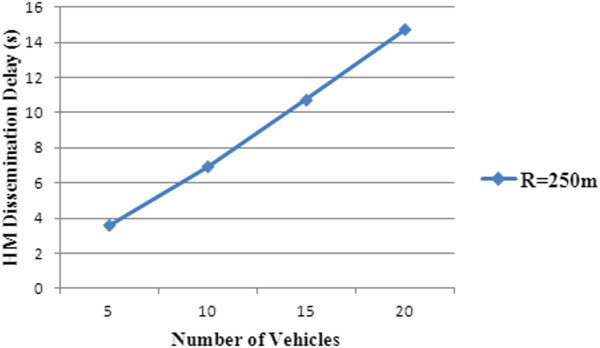
**HM dissemination delay.**

The HM dissemination time is the total elapsed time from sending of hazard messages by the vehicle to the time it is received and verified by the RSU. Figure 11 shows the hazard dissemination time delay plotted against count of vehicles travelling towards the hazard zone.

As the RSU would wait to receive all the hazard messages from the vehicles in the hazard zone during the verification period, the HM dissemination delay shows increases as the number of vehicles in the hazard zone increases.

**Figure 12:**
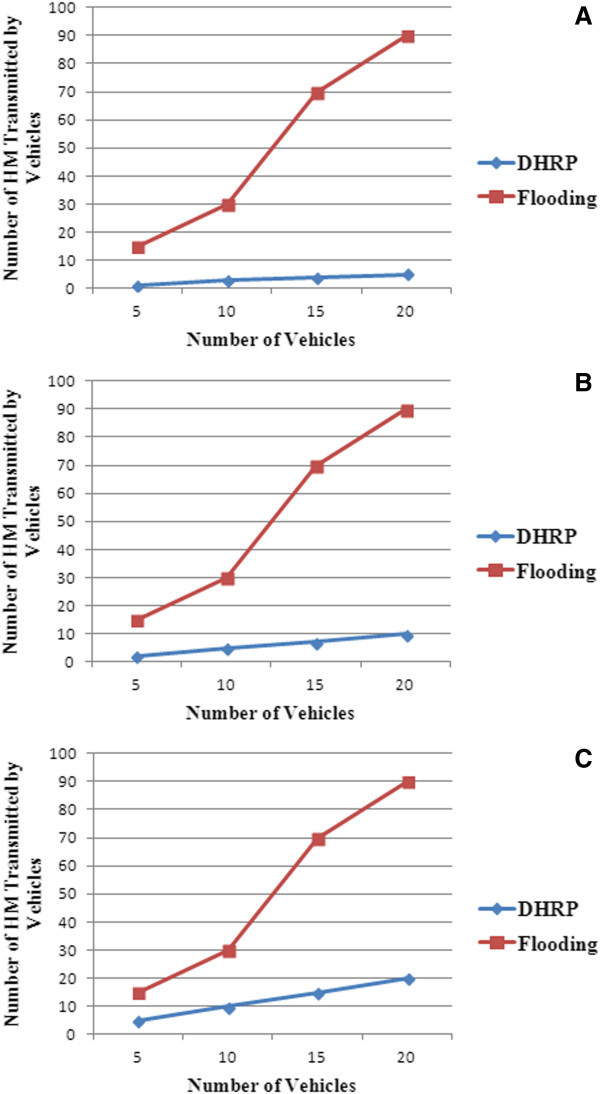
**Number of vehicles in hazard zone. (a)** 25% of vehicles are in hazard zone. **(b)** 50% of vehicles are in hazard zone. **(c)** 100% of vehicles are in hazard zone.

The performance of DHRP protocol was compared with simple flooding protocol with respect to number of packets transmitted and packet loss. In flooding technique, vehicle which detects hazard would broadcast the HM to neighboring vehicles. These vehicles would in turn rebroadcast the same HM to its neighbors. The number of hazard messages transmitted for DHRP and Flooding protocol has been plotted for the assumption that number of vehicles in the hazard zone is 25%, 50% and 100% of the total number of vehicles in the highway and the results are presented in Figure 12(a)–12(c).

In DHRP protocol, only the vehicles in the hazard zone (moving towards the hazard) would transmit the hazard message, but in flooding technique all vehicles that receive the HM would re-broadcast the message. It can be seen from the graph that the number of messages transmitted sharply increases for the flooding technique when the density of the vehicles on the road becomes high.

**Figure 13:**
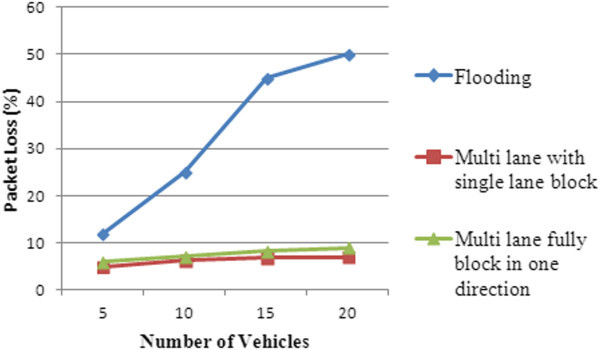
**Packet loss.**

The packet loss for DHRP and flooding protocol has been plotted and the result is presented in Figure 13.

From the figure, it can be noted that the packet loss has increased as vehicle density increases for both the protocols. However, the increase in case of DHRP protocol is very minimal as compared to flooding, as the total number of messages transmitted in the case of DHRP is very much less when compared to flooding.

## Conclusion and future work

In this paper we have discussed and presented DHRP, Direction based Hazard Routing Protocol for transmission of road hazard messages in highways with the use of smart RSUs. RSUs provide a feasible and cost-effective option for disseminating road hazard messages and provide a reliable alternate to V2V communication, especially, in highways where the vehicular traffic is sparse. DHRP would be used for disseminating information about different fixed road hazards such as road blocks, tree fall, boulders on road, and other obstacles to the vehicles approaching the hazardous locations. DHRP carries out selective transmission of hazard message based on the location of hazard and the direction in which the vehicles are travelling. As RSUs would be responsible for disseminating road hazard messages to the vehicles, the network overhead is highly reduced and high reliability can be achieved. The protocol was implemented and tested using SUMO and NS2.33 simulators for transmission range of 250 m. As future work, it is proposed to extend the protocol for RSU deployment with large interspacing and also to highways with varied traffic.
